# Chemotherapy for metastatic colon cancer: No effect on survival when the dose is reduced due to side effects

**DOI:** 10.1186/s12885-018-4380-z

**Published:** 2018-04-23

**Authors:** Stefan Munker, Michael Gerken, Petra Fest, Claudia Ott, Elisabeth Schnoy, Stefan Fichtner-Feigl, Philipp Wiggermann, Martin Vogelhuber, Wolfgang Herr, Christian Stroszczynski, Hans Jürgen Schlitt, Matthias Evert, Michael Reng, Monika Klinkhammer-Schalke, Andreas Teufel

**Affiliations:** 1 Department of Internal Medicine I, University Hospital Regensburg, Franz-Josef-Strauss Allee 11, 95053 Regensburg, Germany; 2 Cancer Center, Institute for quality assurance and health services research, University of Regensburg, Regensburg, Germany; 3 Medical Informatics Unit, University Hospital Regensburg, Regensburg, Germany; 4 Department of Surgery, University Hospital Regensburg, Regensburg, Germany; 5 Department of Radiology, University Hospital Regensburg, Regensburg, Germany; 6 Department of Internal Medicine III, University Hospital Regensburg, Regensburg, Germany; 7 Department of Pathology, University Hospital Regensburg, Regensburg, Germany; 8 MedicDAT GmbH, Regensburg, Germany

**Keywords:** Dose reduction, Cancer, Colorectal cancer, Chemotherapy

## Abstract

**Background:**

5-Fluorouracil (5FU), Folinic acid (FA), and Oxaliplatin (FOLFOX) or 5FU, FA, and Irinotecan (FOLFIRI) are standard regimens for palliative chemotherapy of metastatic colon cancer. Since data showing the influence of dose reduction in palliative treatment are rare, the objective of this single center, retrospective study was to further characterize the influence of dose reduction on efficacy of these therapeutic regimens.

**Methods:**

One hundred nine patients, diagnosed with stage IV colon cancer between 2004 and 2012 and receiving palliative first-line chemotherapy with either FOLFOX or FOLFIRI regimens in our outpatient clinic were analyzed for treatment efficacy. Patients who received dose reductions due to side effects usually received doses of 80% or lower of per protocol dose. Survival data were obtained from the Regensburg Tumor Registry. Survival analysis was performed using Kaplan-Meier statistical analysis and multivariable analysis.

**Results:**

A dose reduction due to side effects was necessary in 46 (42%) patients. Dose reduction was independent of age. Major reasons for dose reduction were neutropenia (30%) followed by polyneuropathy (16%) and diarrhea (14%). Dosage was more often reduced in patients receiving FOLFOX based therapy. Comparison of patients with dose reduction versus patients with full dosage showed no significant difference on overall survival (*p* = 0.430). Subgroup analysis revealed dose reduction in patients with N2 stage disease was associated with improved survival. Patients who underwent dose reduction received more cycles of chemotherapy (13.7 vs. 10.8 cycles) and cumulative dosage was similar in both groups.

**Conclusion:**

Contrary to our expectations, the need to reduce chemotherapy dosage due to side effects does not indicate a worse prognosis in our retrospective analysis. We believe this can in part be explained by better adaption to interindividual pharmacokinetics and longer time of treatment.

**Electronic supplementary material:**

The online version of this article (10.1186/s12885-018-4380-z) contains supplementary material, which is available to authorized users.

## Background

Colon cancer is the third most common cancer and a major cause of morbidity and mortality worldwide [1]. During the last few decades, improvement in therapeutic regimens for advanced colorectal cancer led to a dramatic increase in efficacy, reduction of mortality rates, and improved survival. Upon diagnosis, 20% of newly diagnosed colorectal cancer patients present with metastatic disease with no curative treatment options currently available. Among the chemotherapy regimens considered effective in palliative treatment, Irinotecan or Oxaliplatin in combination with 5-Fluorouracil regimens are standard back bones of current systemic treatment [2, 3].

These chemotherapy regimens were initially tested for efficacy in well-defined study populations not necessarily reflecting average (multimorbid older) patients in real life settings [4, 5]. Clinicians increasingly realize the shortcoming of the initial studies [6] since these mostly included younger patients better able to cope with adverse side effects or toxicities [7]. But especially in elderly and comorbid patients, side effects, organ toxicities and therefore potentially limited survival need to be considered [8]. In clinical practice, these effects are prevented or mitigated by a dose reduction of chemotherapy with the suspected consequence of worse tumor related survival [9]. We therefore believe that there is a need to further investigate the impact of dose reduction of the currently used therapeutic “standard” regimens on survival and side effects in different subgroups. Hence, we performed a retrospective analysis of our patient cohort with advanced stage colorectal cancer patients to assess outcome of reduced chemotherapy dosage.

## Methods

### Study design

A retrospective analysis was performed based on clinical data obtained from the population-based clinical cancer registry at the Regensburg Tumor Center in Eastern Bavaria, Germany. Epidemiological data and survival were investigated in a cohort of colon cancer diagnosed between 2004 and 2012 receiving chemotherapy in the outpatient clinic of the University Medical Center Regensburg. Patients with stage IV colorectal cancer undergoing palliative combination chemotherapy were divided into dose reduction (≤ 80%) and full dosage (100%) groups. We intend to analyze survival time by Kaplan Meier method and to estimate 2- and 3-year survival rates and mean and median survival time. Data collection and retrospective analysis of patient information were anonymized in accordance with the Declaration of Helsinki, and in line with the Bavarian Law of Cancer Registration.

### Background and data collection

The baseline cohort of the present study consisted of patients with the ICD-10-GM (http://www.dimdi.de/static/de/klassi/icd-10-gm/index.htm) diagnosis C18, i.e. a malignant neoplasm of colon. 109 patients with advanced stage colon carcinoma (UICC Stage IV) with histologically confirmed adenocarcinoma of the colon between 2002 and 2012 receiving palliative chemotherapy in our University Medical Center were included in the study. Neuroendocrine tumors were excluded. 3 Patients receiving only 5FU based chemotherapy regimens without Irinotecan or Oxaliplatin were excluded. Prior to palliative chemotherapy, 72 patients received palliative or oncological tumor resection. In this subgroup, the diagnosis of metastasized colon cancer was made during surgery or in follow up examinations. Patients were routinely assessed prior to chemotherapy by a physician trained in oncology. Efficacy evaluation was performed by CT scans in 3-month intervals. Patients who died of cancer unrelated causes and patients with death within one month after start of palliative chemotherapy were censored.

Baseline characteristics are shown in Table 1. For assessing comorbidities a scoring according to the Charlson Score comorbidities index [10] was performed Additional file 1: Table S1. Patients receiving either FOLFOX, FOLFIRI or sequentially both chemotherapy regimens were included in this study. Mode of administration was exclusively i.v.. Chemotherapy dosing values were calculated and registered with a chemotherapy planning software (OnkoDAT®) [11]. A patient was considered receiving reduced dosage of chemotherapy if he received ≥3 cycles of less than 80% of 5FU, Oxaliplatin, or Irinotecan. Survival information were obtained from the Regensburg Tumor Center founded in 1991. The Cancer registry includes epidemiological and clinical data from all consenting patients with malignancies diagnosed and treated in Eastern Bavaria (2.1 million population). All data were extracted, recorded, and fed into a central database by trained personnel. The patients’ life status and disease recurrence were ascertained from clinical reports, death certificates issued by the local public health departments, and the registration offices of the respective residential districts. Data were processed and secured according to the Bavarian Law of Cancer Registration.

### Statistical analysis

Continuous data were described as means, median, minimum, maximum values and standard deviation, and categorical data were expressed as absolute frequencies and relative percentages. Patient characteristics were compared with t-tests for normally distributed continuous data, otherwise by means of non-parametric Mann-Whitney-U- and Chi-square tests for categorical variables. Tumor-specific survival rates (OS) were analyzed from the start of palliative chemotherapy until the event of death or last patient contact. Survival rates of patients with and without dose reduction were described by Kaplan-Meier analysis. Survival differences were tested for statistical significance by the two-sided Log Rank in Kaplan-Meier analyses; the level of significance was set to 0.05. The follow-up period and survival times were right censored using December 31, 2012 as cut-off date. To determine the influence of further co-variables on overall survival, we performed univariable and multivariable regression analysis using Cox proportional hazard models. In multivariable analysis, the hazard ratio (HR) was adjusted for the co-variables age, sex, TNM status, grading, lymph vessel invasion, vein invasion, Charlson score for comorbidities, surgery and further chemotherapy, i.e. number of lines and regimens. Again, a two-sided *p*-value of 0.05 was considered to indicate statistical significance. Hazard ratios and corresponding 95% confidence intervals (CI) were calculated and considered statistically significant when the CI excluded 1.0. All analyses were performed using IBM SPSS Statistics, version 23.0.

## Results

### Reduction of chemotherapy dosage

Among 109 patients 67% (73/109) of the patients received both (FOLFOX and FOLFIRI) regimens sequentially. The majority 78% (57/73) began with FOLFOX and changed during the course of disease to FOLFIRI, 21% (23/109) of the patients received exclusively FOLFOX, whereas FOLFIRI chemotherapy regimens were used exclusively in 12% (13/109) of patients. 43% (47/109) received additional treatment with a biological agent.

Upon discretion of the treating physician, in most cases dosage was reduced to 80% of the protocol dosage. We therefore used reduction to 80% of protocol dosage as a cut off to investigate the clinical effectiveness of standard chemotherapy in patients receiving reduced dosage.

In 42% (46/110) of our patients a dose reduction to 80% or less was performed during the course of chemotherapy. Those 46 patients who received a dose reduction, received in average 14 chemotherapy cycles in our clinic (Mean: 13.7 cycles; + − 12.3 cycles). Patients continuing on protocol chemotherapy doses, received 10.5 cycles (stdv 10.8, *p* = 0.149). In more detail, the majority of chemo cycles (72%, median: 81%; stdv: 27%) in these patients were applied with reduced dosage. Average cumulative dosage and dose intensities for 5-Flourouracil, Irinotecan and Oxaliplatin were calculated for full dosage and dose reduction subgroups (Table 2). As expected, relative dose intensities were significantly different. Even though a trend to a higher cumulative dosage can be observed, t-test revealed no significant differences for cumulative dosages.

### Dose reduction in subgroups – analysis of distribution

In preparation to compare survival rates, we compared the distribution of the co-variables age, sex, TNM status, grading, lymph vessel invasion, vein invasion, Charlson score for comorbidities, surgery and further chemotherapy, i.e. number of lines and regimens in the dose reduction and full dosage group.

No significant difference was seen except for lymph vessel and vein invasion, CTX regimen and adverse side effects, when Chi-square test for independence was performed. As expected, subgroups of patients suffering from severe symptoms/side effects related to chemotherapy such as diarrhea (*p* < 0.001), PNP (*p* < 0.001), or neutropenia (*p* = 0.02) received dose reduction almost exclusively. Detailed summary of the distribution of dose reductions in different subgroups is given in Table 1.

**Table 1:** Patient characteristics according to dosage reduction and in total

		Dosage reduction						
		Yes (<=80% dosage)		No (=100% dosage)		Total		T-test Chi^2^
		*N*	(%)	*N*	(%)	*N*	(%)	*p* value
Age	Mean/Median	56.5	59.0	58.4	58.0	57.6	59.0	.440
	< 65	32	69.6%	42	66.7%	74	67.9%	.749
	> = 65	14	30.4%	21	33.3%	35	32.1%	
Sex	Male	29	63.0%	46	73.0%	75	68.8%	.267
	Female	17	37.0%	17	27.0%	34	31.2%	
T stage	T3	17	37.0%	23	36.5%	40	36.7%	
	T4	22	47.8%	22	34.9%	44	40.4%	.205
	Tx	7	15.2%	18	28.6%	25	22.9%	
N stage	N0	4	8.7%	5	7.9%	9	8.3%	
	N1	14	30.4%	16	25.4%	30	27.5%	.796
	N2	18	39.1%	23	36.5%	41	37.6%	
	Nx	10	21.7%	19	30.2%	29	26.6%	
Grading	G2	27	58.7%	40	63.5%	67	61.5%	
	G3	18	39.1%	19	30.2%	37	33.9%	.419
	Gx	1	2.2%	4	6.3%	5	4.6%	
L stage	L0	6	13.0%	7	11.1%	13	11.9%	
	L1	23	50.0%	15	23.8%	38	34.9%	.010
	Lx	17	37.0%	41	65.1%	58	53.2%	
V stage	V0	13	28.3%	11	17.5%	24	22.0%	
	V1	14	30.4%	10	15.9%	24	22.0%	.030
	Vx	19	41.3%	42	66.7%	61	56.0%	
Charlson Comorbidity Score	6	32	69.6%	44	69.8%	76	69.7%	
	7	7	15.2%	13	20.6%	20	18.3%	
	8	6	13.0%	4	6.3%	10	9.2%	.336
	9	0	0.0%	2	3.2%	2	1.8%	
	10	1	2.2%	0	0.0%	1	0.9%	
Oncological resection/Surgery	Yes	35	76.1%	37	58.7%	72	66.1%	.059
	No	11	23.9%	26	41.3%	37	33.9%	
No. of CTX lines	1	11	23.9%	17	27.0%	28	25.7%	
	2	23	50.0%	34	54.0%	57	52.3%	
	3	11	23.9%	11	17.5%	22	20.2%	.587
	4	0	0.0%	1	1.6%	1	0.9%	
	5	1	2.2%	0	0.0%	1	0.9%	
Biological	Yes	21	45.7%	26	41.3%	47	43.1%	.648
	No	25	54.3%	37	58.7%	62	56.9%	
CTX regimen sequence	FOLFOX- > FOLFIRI	30	65.2%	27	42.9%	57	52.3%	
	FOLFIRI- > FOLFOX	3	6.5%	13	20.6%	16	14.7%	.044
	FOLFOX	10	21.7%	13	20.6%	23	21.1%	
	FOLFIRI	3	6.5%	10	15.9%	13	11.9%	
	Total	46	100.0%	63	100.0%	109	100.0%

**Table 2:** Dose intensity and cumulative dosage for the dose-reduction and full dosage subgroups

	Substance	Dose group	Mean in mg	standard deviation	*p*-value
Cumulative Dosage	5-Fluorouracil	100%	54,934	55,617	0.42
		< 80%	64,321	61,824	
	Oxaliplatin	100%	1046	852	0.34
		< 80%	1245	976	
	Irinotecan	100%	2861	3295	0.77
		< 80%	3099	3296	
Dose intensity	5-Fluorouracil	100%	5383	1450	0.002
		< 80%	4547	1178	
	Oxaliplatin	100%	173	31	0.03
		< 80%	156	35	
	Irinotecan	100%	311	74	0.04
		< 80%	274	65	

*P*-values were calculated with t-test

**Table 3:** Reasons for dose reduction

Distribution of adverse effects	*N*	%
Leukopenia	20	30%
Polyneuropathy	11	16%
Diarrhea	10	14%
Worsening of general condition	6	9%
Hand-foot-syndrome	5	7%
Thrombocytopenia	5	7%
Hyperemesis	3	4%
Elevated bilirubin	2	3%
Deterioration of kidney function	1	1%
Mucositis	1	1%
Not specified	5	7%

**Table 4:** Results of univariable and multivariable Cox-regression for survival according to dosage reduction

				95% CI for HR	
		*p* value	Hazard ratio	Lower	Upper
Univariable Cox-regression					
Dosage reduction	No		1.000		
	Yes	.431	.841	.547	1.294
Multivariable Cox-regression					
Dosage reduction	No		1.000		
	Yes	.600	.861	.492	1.506
Age	continuous	.118	.981	.958	1.005
Sex	Male		1.000		
	Female	.446	1.264	.692	2.310
T stage	T3		1.000		
	T4	.229	1.541	.761	3.117
	TX	.890	.916	.265	3.170
N stage	N0		1.000		
	N1	.981	.988	.345	2.824
	N2	.713	1.225	.415	3.615
	NX	.927	.922	.161	5.266
Grading	G2		1.000		
	G3	.302	1.354	.761	2.407
	GX	.458	.576	.134	2.474
L stage	L0		1.000		
	L1	.726	1.215	.408	3.620
	LX	.289	.469	.116	1.901
V stage	V0		1.000		
	V1	.872	.920	.334	2.531
	VX	.164	2.620	.675	10.165
Charlson Score	6		1.000		
	7	.750	.892	.443	1.797
	8	.909	.954	.426	2.138
	9	.609	1.605	.262	9.822
Oncological resection/Surgery	No		1.000		
	Yes	.227	.483	.149	1.570
No of CTX lines	continuous	.797	1.053	.712	1.556
Biological CTX	No		1.000		
	Yes	.178	.663	.364	1.207
CTX regimen	FOLFOX- > FOLFIRI		1.000		
	FOLFIRI- > FOLFOX	.691	1.177	.527	2.627
	FOLFOX	.001	3.601	1.656	7.827
	FOLFIRI	.921	.945	.309	2.892

**Table 5:** Subgroup analysis. Comparison of the survival after dose reduction versus full dosage in different subgroups analyzed by Kaplan-Meier procedure and Log-Rank test (Chemotherapy (CTX))

		Dosage reduction		Log-rank
		Yes (≤ 80%)	No (100%)	
		Mean (Median) survival in months		*p*-value
Age	< 65	22.0 (14.6)	17.1 (12.0)	.170
	≥65	18.8 (14.9)	21.2 (15.8)	.764
Sex	Male	23.5 (16.0)	18.6 (14.9)	.339
	Female	17.2 (13.1)	17.4 (12.7)	.170
T stage	T3	27.8 (26.4)	25.8 (14.9)	.525
	T4	15.6 (13.8)	14.1 (12.7)	.960
	Tx	24.3 (10.4)	14.1 (7.7)	.273
N stage	N0	25.9 (23.6)	30.5 (14.9)	.851
	N1	20.7 (13.9)	24.1 (12.3)	.669
	N2	21.3 (26.4)	13.3 (16.7)	.024
	Nx	20.4 (16.0)	13.2 (7.7)	.294
Grading	G2	24.1 (17.8)	22.0 (16.1)	.570
	G3	17.5 (14.5)	10.6 (10.4)	.062
	Gx	26.8 (26.8)	25.5 (3.0)	.808
L stage	L0	19.7 (17.8)	30.7 (21.0)	.541
	L1	23.3 (13.9)	16.9 (17.5)	.485
	Lx	18.1 (14.8)	16.3 (12.0)	.561
V stage	V0	28.8 (23.6)	26.9 (21.0)	.828
	V1	20.3 (13.9)	15.7 (16.7)	.311
	Vx	16.9 (14.5)	15.9 (11.8)	.671
Charlson Score for Comorbidities	6	20.6 (14.8)	19.6 (12.0)	.424
	7–10	23.3 (15.0)	19.6 (16.1)	.632
Oncological resection/Surgery	Yes	22.1 (14.9)	23.8 (17.5)	.997
	No	19.6 (12.9)	12.9 (7.6)	.301
No. of CTX lines	1	10.1 (10.4)	8.4 (3.0)	.565
	2	23.1 (17.9)	23.5 (15.8)	.896
	3	24.7 (23.6)	16.6 (12.3)	.238
Biological CTX	Yes	29.4 (26.8)	23.5 (11.8)	.371
	No	13.2 (13.1)	14.6 (14.9)	.823
CTX regimen sequence	FOLFOX- > FOLFIRI	22.0 (20.2)	26.5 (17.5)	.425
	FOLFIRI- > FOLFOX	12.3 (12.3)	16.4 (14.9)	.631
	FOLFOX	9.7 (12.9)	7.7 (3.6)	.829
	FOLFIRI	62.3 (90.7)	13.0 (3.9)	.300

### Reasons for dose reduction

The most common reason for dose reduction of chemotherapy was neutropenia (30%). Other common side effects leading to dose reduction were polyneuropathy (16%) and diarrhea (14%). Polyneuropathy was generally due to treatment with Oxaliplatin (10 patients, 14%). Diarrhea was observed at similar rates in both Oxaliplatin (6/48 patients, 13%) and Irinotecan treated patients (4/30 patients, 13%). Less common reasons for dose reduction included mostly symptom-related causes, hyperemesis, worsening of general condition, hand foot syndrome and mucositis. A complete overview of the reasons for dose reduction of chemotherapy is shown in Table 3.

### Survival in dose reduced versus full dose 5-FU based regimens

**Fig. 1:**
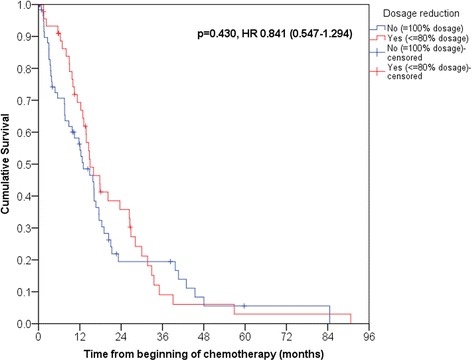
Kaplan Meier analysis of survival in patients with reduced and full dosage of standard chemotherapy back bone (FOLFIRI, FOLFOX). Survival analysis showed no difference in survival (*p* = 0.430, Log Rank)

In terms of tumor-specific survival, we did not observe any differences between patients receiving full dose and reduced dose chemotherapy (Log Rank, *p* = 0.430) **(**Fig. 1**)**. Median survival for patients receiving full dosage was 13.0 months (Mean 19.1), for patients with dosage reduction 14.9 months (Mean 21.2). Two-year survival was 19.5% (full dosage) vs 35.8% (reduced dosage). Three-year survival rate of patients with full dose and reduced dose chemotherapeutic treatment was 19.5% and 9.1%. A univariable Cox regression rendered a hazard ratio of 0.841 (95% CI 0.547–1.294; *p* = 0.431) for the dose reduction group versus full reduction. After adjustment for age, sex, TNM status, grading, lymph vessel invasion, vein invasion, Charlson score for comorbidities, surgery and further chemotherapy, i.e. number of lines and regimens in a multivariable Cox regression analysis the hazard ratio for patients with dose reduction was 0.861 (95% CI 0.492–1.506; *p* = 0.600) Table 4.

### Efficacy – subgroup analysis

We performed a subgroup analysis to identify potential subgroups in which reduction of chemotherapy might be beneficial or harmful. Table 5 summarizes survival in several subgroups with respect to dose reduction. In most subgroups, no significant differences in survival were observed. Unexpectedly, in the stage N2 lymph node subgroup with 41 patients dose reduction was associated with improved survival (Log Rank *p* = 0.024).

## Discussion

Over the past decades a considerable progress has been made in the management of colorectal cancer by medical oncologists. The studies leading to these advances were predominantly performed on young and healthy populations; hence the common practice of dose reduction in elderly or frail patients was not a primary issue. Thus, these studies were mainly performed on a subgroup not suffering from relevant co-morbidities and being in good performance status.

In recent years, the need to investigate real life populations and the common practice of dose reduction has been recognized by the scientific community. However, (retrospective) studies investigating the effects of dose reduction in a palliative setting have been published but are still sparse. In 2001, a retrospective analysis of patients with Stage II-III colon cancer demonstrated that in an adjuvant setting 5-FU based chemotherapy may be safely administered in the elderly, but this study did not elaborate the dose reduction needed [12]. In 2011 Langley et al. published the Focus II study, a first randomized controlled trial including only the frail and old patients with colorectal cancer. This study incorporated primary dose reduction as the standard treatment for all treatment arms. However, it was not designed to investigate whether dose reduction itself could be performed if required without affecting PFS or OS [13].

In order to evaluate whether dose reduction has an effect on survival in patients with advanced colorectal cancer and suffering from side effects under standard treatment dose, we performed this retrospective analysis of such patients in our outpatient clinic. In our current study, we chose a cutoff for dose reduction of 80% since in our experience a dose reduction to 80% is a commonly performed reduction in case of adverse reactions. Since only very few patients received further dose reduction (*n* = 4), we chose not to include these patients as a separate group with dose reduction of ≤50% but rather included these patients in the group of patients with reduction of chemotherapy dosage. Thus, the overall average dose reduction in this patient group was even more than 20%. In these patients, a dose reduction was applied to the majority of cycles (72%), emphasizing that this group predominantly received a reduced chemotherapy dosage throughout the course of therapy.

In daily clinical routine, clinicians are more likely to reduce the dose of elderly patients. Therefore, one might assume that in the elderly (> 65 years) in our collective dose reduction would be more common. However, dose reduction was evenly distributed among younger and elderly patients and thus independent of age. Also, additional subgroup analysis (T-stage, N-stage, M-stage, gender, and chemotherapy regimen) showed an even distribution of dose reduction.

Since clinical trials showed the effectiveness of per protocol chemotherapy, in theory a reduced dosage of chemotherapy would be expected to affect survival, which has also been confirmed by several publications for other entities [9]. It is therefore common belief, that dose reduction should be avoided. Additionally, clinicians choose a dose reduction often due to symptom-related causes or deterioration of laboratory findings. Many of these reasons (recurring neutropenia, deterioration of general condition) are associated with a poor clinical outcome. Therefore, one might assume, that dose reduction is more common in patients with clinical features suggesting a poor prognosis. Interestingly, statistical analysis of our data showed that a moderate dose reduction does not affect survival significantly.

In a recently published manuscript, the influence of the relative dose intensity (RDI, of adjuvant 5FU and Oxaliplatin combination treatment in veterans with Stage III colon cancer had been further investigated. Aspinalli et al. showed that a major reduction of RDI (< 70%) to be associated with worse overall survival in this patient group [14]. In addition to the bias of the patient group which consisted mainly of male veterans, there was a bias towards dose reduction in the frail and elderly. In comparison with our study also the magnitude of dose reduction was more pronounced.

When Oxaliplatin was introduced to the treatment of CRC, a retrospective analysis of three phase II studies of pretreated colorectal cancer showed that higher dose intensity leads to improved survival [15]. In contrast to our study, these study populations had been pretreated and in two out of the three studies patients’ inclusion age was limited to younger patients. In another more recent study, Nakayama et al. showed that a dose reduction in metastasized CRC led to poorer survival of the respective (Irinotecan) patient group. A comparison with our study population is difficult, since per protocol Irinotecan dosage in Japan is already 12% lower than in the western countries and further dose reduction adds up to even more pronounced dose reductions. For the patient group receiving Oxaliplatin only PFS was significantly associated to RDI [16].

Nevertheless, in clinical practice, patients often experience side effects and need dose reduction. For these patients, our data in treatment-naive patients suffering from stage IV colorectal cancer, suggest that a moderate dose reduction does not necessarily result in less efficacy. Until now, only limited data were reported on this issue, which we believe to be of high clinical relevance. Thus, we suggest further randomized studies potentially leading to more personalized treatment strategies depending on tolerance of treatment and co-morbidities and a more side effect oriented approach on chemotherapy dosing.

## Conclusion

In our group of patients with colorectal cancer treated in a palliative setting, the need for a moderate reduction of chemotherapy due to side effects has no measurable effect on survival. This may be in part due to better adaption to interindividual pharmacokinetics and to a longer treatment of patients with reduced chemotherapy dosage if side effects cause dose reduction.

## Additional file

Additional file 1: **Table S1.** Summary of comorbidities. (DOCX 13 kb)
