# Supplementary material for: Chemotherapy for metastatic colon cancer: No effect on survival when the dose is reduced due to side effects
Source: BMC Cancer. 2018 Apr 23;18:455. doi: 10.1186/s12885-018-4380-z (PMC5913883; doi:10.1186/s12885-018-4380-z)
Supplement: Supplementary file 1 — Table S1. Summary of comorbidities. (DOCX 13 kb) [file 12885_2018_4380_MOESM1_ESM.docx]

Additional file 1 **Table S1** Summary of comorbidities

| **Disease** | **Charlson Score Points** | **Number of patients** |
| --- | --- | --- |
| Metastatic disease with no concurrent comorbidity | 6 | 56 |
| Arterial Hypertension | 0 | 33 |
| Diabetes mellitus | 1 | 12 |
| Myocardial infarction | 1 | 7 |
| Chronic renal disease | 2 | 6 |
| Hemiparesis | 2 | 4 |
| Heart failure | 1 | 3 |
| Chronic pulmonary disease | 1 | 2 |
| Gastric Ulcers | 1 | 2 |
| Collagenosis | 1 | 1 |
